# Limiting the Growth of Water-Soluble, Monolayer-Protected Quantum Dots

**DOI:** 10.1155/2018/3164347

**Published:** 2018-07-02

**Authors:** Ava E. Conner, Veronica Gordillo-Herrejon, Sonia C. Francone, Emily A. Shriner, Fernando E. Acosta, Nathan D. Barnett, Deon T. Miles

**Affiliations:** Chemistry Department, University of the South, Sewanee, TN 37383, USA

## Abstract

The growth and solubility of quantum dots (QDs) are important factors that must be examined before these nanoparticles are incorporated into a variety of potential applications. In this work, monolayer-protected CdSe QDs surrounded by water-soluble thiols were prepared using various cadmium salts. The use of a variety of cadmium salts did not have a significant impact on the spectral properties of the CdSe QDs. CdSe QDs were synthesized at rather low temperatures (< 0°C), resulting in slow nanoparticle growth upon subsequent heating of the reaction mixture. The effect of multiple drying and redissolving cycles of the QD samples was examined. The effect of heating temperature on QD growth was studied, with more rapid nanoparticle growth associated with higher temperatures. The results show that QDs can be synthesized at low temperatures and their subsequent growth can be controlled during the heating process.

## 1. Introduction

Nanometer-sized fluorescent semiconductor nanocrystals, also known as quantum dots (QDs), have been the focus of significant research efforts for both their physical properties and the possible potential applications that are a consequence of these properties. The most striking physical behavior of these materials is their tendency to display an intense luminescence, with the wavelength determined at least in part by the size of the nanoparticles. Applications include such diverse possibilities as solid-state lighting, photovoltaic materials, optoelectronic devices, and pH sensors [1–4]. These nanomaterials are fabricated from a host of compound semiconductors, including II-VI (CdSe, ZnO) and III-V (GaAs, InP) materials. A seminal paper on the synthesis of high-quality CdSe QDs was reported by the Bawendi group in 1993 [5]. Their procedure was based on the pyrolysis of organometallic reagents by injection into a hot coordinating solvent. These QDs were organic-soluble, which limited their application to biologically based situations. The QDs synthesized initially by the Bawendi group used starting materials that are considered toxic. In an effort to employ principles associated with green chemistry, CdSe nanoparticles were synthesized by the Peng group in 2001 [6]. In their syntheses, cadmium acetate was used as the cadmium precursor, and fatty acids were used as the solvent. These materials are not toxic or pyrophoric, and their cost is lower than previous materials used in CdSe QD synthesis. However, these QDs are soluble in organic solvents, which limits their use in biological systems.

For most semiconducting nanoparticles, the QD surface is passivated during nanoparticle synthesis with capping ligands. For example, alkyl thiols are capping ligands that can both limit growth and prevent aggregation of the QDs [7]. The capping ligands can also be used to provide additional functionality to the system and/or to control the solubility of the QDs. For example, QD-based pH sensing devices were developed by incorporating 1,3-oxazine moieties into the passivating thiol ligands [8, 9]. Not surprisingly, the use of alkyl thiols produces QDs that are soluble in organic solvents, while use of thiols with polar functional groups results in aqueous solubility [7]. Water-soluble QDs are more amenable for use in biological applications and can be manipulated in less hazardous solvents than their organic counterparts.

There are several approaches to prepare water-soluble QDs found in the literature [10, 11]. One example of preparing water-soluble QDs quickly involved the use of hydrazine (a highly toxic compound) during the synthesis [12]. Another approach involves starting with organic-soluble versions of the QDs, then performing a place-exchange reaction with dithiocarbamates to make the nanoparticles soluble in water [13]. Another research group uses a multidentate biopolymer to prepare QDs that are water-soluble [14]. Typically, the motivation for preparing water-soluble QDs using these new techniques is to (1) reduce the hazards associated with established techniques, (2) improve the biocompatibility of the nanomaterials, and (3) reduce the potential cytotoxicity associated with the use of heavy metals. Here, we employ a modified version of an established synthetic method [7] that uses less hazardous metal and chalcogenide precursors than the popular organometallic method, which is based on high-temperature thermolysis of the QD precursors in inert atmospheres [5]. In addition to less hazardous materials being used, inert atmospheric conditions are not required for this QD synthesis; it can be performed under ambient conditions. The QDs produced are composed of a semiconductor CdSe core that is stabilized by a surrounding layer of water-soluble thiols.

To realize the potential applications of QDs, it is necessary to (1) control the growth and aggregation of the QDs and (2) develop robust methods for controlling the solubility of the QDs in various solvents. For example, being able to grow QDs of a particular size is important in their incorporation into digital displays, where small differences in size are related to color acuity. Concerning the solubility of the QDs, it is rather straightforward that QDs that are water-soluble are likely candidates for biological applications (e.g., fluorescent biosensors). Therefore, it is important to study the properties of these nanoparticles involving growth and solubility at different temperatures and solvents to improve and discover more applications for QDs. Controlling the QDs growth during synthesis and examining their solubility are the focal points of this report.

## 2. Materials and Methods

### 2.1. Nanoparticle Synthesis

Water-soluble, monolayer-protected QDs were prepared using a modification of a previous synthesis [7]. Typically, the QDs (2.4:1 thiol:metal ratio) were synthesized by combining 2.35 mmol cadmium salt (anions = perchlorate, acetate, chloride, nitrate, or sulfate) and 5.7 mmol water-soluble thiol (mercaptosuccinic acid, glutathione, or cysteine) in 125 mL of reverse osmosis (RO) water. The pH was adjusted by the addition of 1 M NaOH with stirring to pH 11 (Scientific Instruments IQ240 pH meter). It is noted that the solution may become slightly turbid during the pH adjustment, but a clear solution is an indication to proceed to the next step. The clear solution was transferred to a three-neck flask and deaerated for 30 min with N_2_ gas while being held at a specified temperature (typically 0°C). For reduced temperature studies, the reaction mixture was either immersed in an isopropanol solvent bath that was temperature-controlled with an immersion cooler (ThermoNeslab, Model CC-100) or an ice-salt bath. In a separate three-neck flask, H_2_Se gas was generated by reacting Al_2_Se_3_ (0.2 g, 0.46 mmol) with 0.5 M H_2_SO_4_ (18 mL) under a N_2_ atmosphere. The H_2_Se gas was bubbled through the metal salt/thiol solution for 5-20 min with the use of a stainless steel cannula. In most instances, the addition of H_2_Se gas resulted in a change in color of the reaction mixture. To increase the particle size, the QD solution was refluxed under open-air conditions with a condenser. Once the QD solution became turbid, which is an indication of QD aggregation, refluxing was discontinued. Aliquots of the QD solution were taken at either timed intervals or when the mixture changed color. As a convention, t_HEAT_ = 0 was considered to be the time at which heat was first applied to the QD solution, which is not the same time that refluxing of the reaction mixture begins. In one part of this study, the temperature was not set to reflux conditions but rather at several temperature intervals. Samples were purified using size-exclusion chromatography (SEC) as described below. All QD solutions were stored at 4°C. Alcohol-soluble, monolayer-protected QDs were prepared in a similar fashion as the water-soluble QDs. The differences in the syntheses include the solvent used for the metal salt-thiol mixture and how the pH of this mixture is adjusted. The mixture was dissolved in 125 mL of alcohol (methanol or ethanol). The pH was adjusted by the addition of* N*,*N*-diisopropylethylamine (DIPEA) with stirring to pH 9. The remainder of the procedure is the same as with the water-soluble QDs. For the solubility study, QD samples were rotary evaporated using a water bath heated up to 80°C for about 30 min. After the majority of the solvent was evaporated, any remaining solvent was removed using a vacuum desiccator. The QDs were subsequently redissolved in another suitable solvent.

### 2.2. Size-Exclusion Chromatography

For SEC purification, a glass chromatography column was filled with Sephadex G-75 (Sigma, 40-120 *μ*m beads, 3-80 kDa fractionation range) which was used as the stationary phase. The stationary phase was prepared by combining 10 g of Sephadex with 200 mL RO water. Approximately 1 mL of each QD solution was run through the SEC column using RO water as the eluent. During an SEC run, each eluate (approximately 5 mL) was collected and sequentially numbered. To help remove thiols and unreacted material from the column between runs, the SEC column was flushed with 0.2 M NaOH (25 mL).

### 2.3. Spectroscopic Methods

A Hewlett-Packard 8453 UV-visible spectrophotometer was used to determine the electronic absorption properties of the QD solutions. A Jobin Yvon Fluoromax-P fluorimeter was used to study the luminescent properties of the QD solutions. Samples for analysis were prepared by placing approximately 5-10 drops (< 1 mL) of the QD solution into a 1-cm quartz cuvette (Fisher) and filling the cuvette with RO water. Typically, an excitation wavelength of 350 nm was applied for fluorescence measurements. The spectral properties of all QD samples were analyzed at ambient temperatures. The determination of fluorescent quantum yield was performed using a standard method [15, 16]. Rhodamine 6G (in H_2_O, Φ = 0.95) was used as the standard for the analysis. The absorbance value of a QD sample at 348 nm was adjusted from 0.10 to 0.01 absorbance units. The integrated fluorescence area was measured from 400 to 800 nm for each of the dilutions. Quantum yield values for QD solutions ranged from 4 to 8%, which is lower than observed with other water-soluble QD systems [17–19].

## 3. Results and Discussion

### 3.1. Alternative Reagents

In the established synthesis of water-soluble QDs, the metal salt used is typically a metal perchlorate hydrate, which is subsequently mixed with a water-soluble thiol (which is an organic compound) and heated under ambient conditions [7]. A significant concern associated with this synthesis is the heating of a perchlorate-organic mixture, which could lead to fire or explosion. To avoid this unsafe situation, the QD synthesis was modified. The first modification involved using commercially available cadmium salts, in particular cadmium acetate, cadmium chloride, cadmium nitrate, and cadmium sulfate.

**Figure 1:**
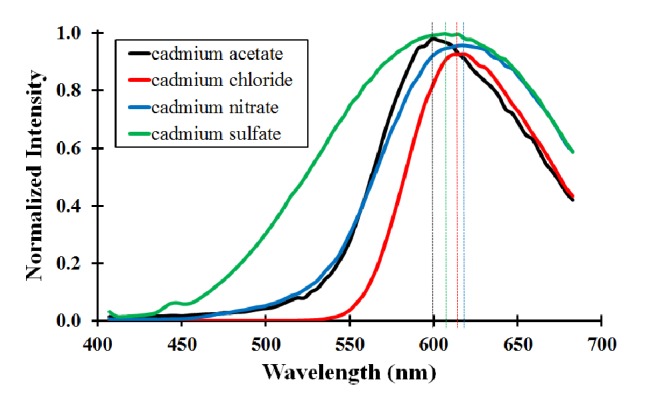
Fluorescence spectra of mercaptosuccinic acid-modified CdSe QDs. Different metal salts were used, with corresponding *λ*_MAX_ values (acetate = 605 nm [fwhm = 111 nm], chloride = 610 nm [fwhm = 134 nm], nitrate = 617 nm [fwhm = 108 nm], and sulfate = 612 nm [fwhm = 176 nm]).

From Figure 1, there appears to be no major differences observed in the fluorescent properties of the as-prepared CdSe QDs due to the type of cadmium salts. The emission *λ*_MAX_ values range from 599 nm (cadmium acetate) to 617 nm (cadmium nitrate). While these differences in emission *λ*_MAX_ values are not significant, the differences in the full width at half maximum (fwhm) values were investigated closely. The fwhm values of the emission spectra provide some indication of the polydispersity of the QD sample. Generally, a larger fwhm value means that there is a larger size distribution of the nanoparticles in the sample. In this set of QDs made from alternative cadmium salts, the major difference between the salts is naturally the type of anion that is present. Considering the properties of the anions, only one of them is doubly charged (SO_4_^2–^), while the others are singly charged. The sulfate ion also has the largest hydrodynamic radius (0.242 nm) compared to the other anions (acetate = 0.231 nm, chloride = 0.180 nm, and nitrate = 0.177 nm) under investigation [20]. The combination of large anion size and higher charge has an impact on the polydispersity observed in the CdSe QDs made from cadmium sulfate. The charge of the anion is probably more significant than the size, considering that those CdSe QDs that are made from cadmium acetate are not as polydisperse, even though the hydrodynamic radius of the acetate ion is comparable to that of the sulfate ion.

**Figure 2:**
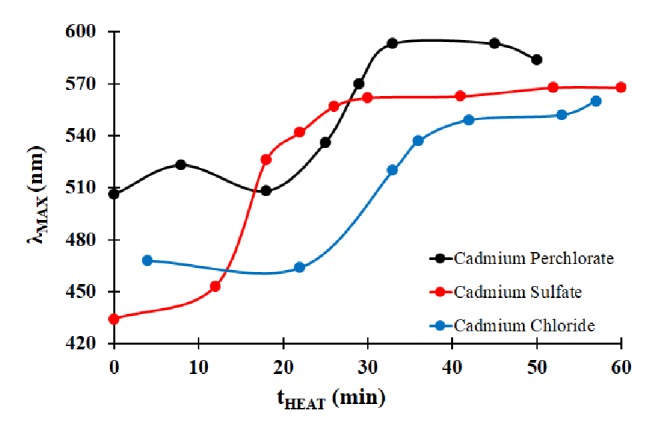
Effect of heating time on fluorescence emission properties of cysteine-modified CdSe QDs using different metal salts.

The effect of heating on the spectral properties of CdSe QDs using different cadmium salts was studied as well. In Figure 2, the comparison of the fluorescence emission properties is made between three different cadmium salts. The growth dynamics are similar to those observed in a previous study, with an initial slow growth period, then a short period of rapid growth before limited growth at extended heating times [21, 22]. From Figure 2, the change in *λ*_MAX_ over a one-hour period was between 80 nm and 100 nm. The changes in spectral properties of the QDs were similar for each of these cadmium salts. There is little difference between the resulting nanoparticles that are made from these different cadmium salts.

### 3.2. Low Temperature Synthesis

Quantum dots made using the popular organometallic method are usually synthesized at very high temperatures (up to 350°C) and under inert atmosphere. The nanoparticles that are produced from this synthetic method usually grow very quickly and are monodisperse. However, from using this method, it is not clear how much control is possible with the growth of the nanoparticles. One method to slow down the growth of the QDs that we investigated was to reduce the temperature of the reaction synthesis. There are several examples of previous work in the area of low temperature synthesis of QDs [23–28]. However, all of the previous work was done using organic-soluble QDs and at temperatures above 0°C. Here, we examined water-soluble QDs that were synthesized at temperatures below 0°C.

If the metal salt-thiol reaction mixture is combined with the chalcogenide source (H_2_Se) at low temperature (< 0°C), then the growth of the QDs will be slowed upon subsequent refluxing. With the typical reaction being done in water, in order to reduce the temperature below 0°C, the solvent used for the synthesis must be changed. The ideal candidates for solvents are in the family of alcohols, in particular methanol (freezing point = –97°C) and ethanol (f.p. = –114°C). With this change in solvent, the manner in which the pH was adjusted was altered as well. Instead of using sodium hydroxide to increase the pH of the reaction mixture in organic solvent, an organic base (DIPEA) was used to adjust the pH to alkaline conditions. It should be noted that the reaction mixture was held at the reduced temperature for up to 1 hour while it was being degassed with N_2_ and subsequently mixed with H_2_Se gas. The reaction mixture was then heated to reflux conditions, with aliquots taken periodically for spectral analysis.

**Figure 3:**
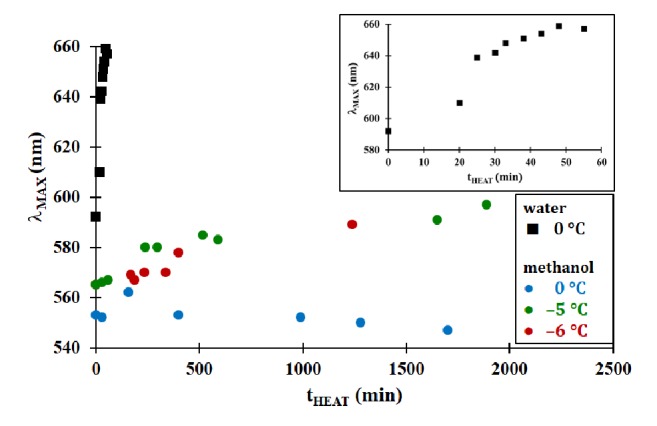
Effect of heating time on the fluorescence emission properties of mercaptosuccinic acid-modified CdSe QDs synthesized at various temperatures. Inset is the expanded water data for clarity.

As seen in Figure 3, the growth of the CdSe QDs in methanol is dramatically slower than in water, with a change in *λ*_MAX_ of less than 30 nm over an extended time period of refluxing (about 30 hours). This change in *λ*_MAX_ is significantly less than that observed in water over a much shorter time period. The change in *λ*_MAX_ for the water-based reaction mixture was roughly 60 nm over a one-hour period of refluxing. However, the temperature of the organic-based reaction mixture was similar to that of the water-based reaction. It is unlikely that this small reduction in temperature would have a substantial effect in the growth properties of the nanoparticles. An experiment was performed to compare only the change in solvent for the reaction mixture and maintain the same temperature (0°C). From Figure 3, it is clear that there was more rapid growth in the size of the QDs in water compared to methanol at the same temperature. The results from this comparison were that the organic solvent plays a significant role in limiting the growth of the QDs. And while the reduction in temperature can have an effect on nanoparticle growth, the change in solvent appears to have a larger effect on the growth dynamics.

### 3.3. Quantum Dot Solubility

One important aspect to investigate before incorporating nanoparticles into potential applications is their solubility. There are a number of factors that affect the solubility of QDs, including size [7], pH [29–31], and capping agents [12, 32]. The monolayer-protected QDs prepared in this study are water-soluble. However, they are soluble in a few organic solvents, specifically the short chain alcohols methanol and ethanol. When attempting to dissolve these nanoparticles in longer chain alcohols, such as isopropanol, the size of the QDs has an impact on solubility. Larger QDs tend to be insoluble in isopropanol, as observed in previous work [7]. Another interesting aspect about these QDs is that since they are surrounded by a protecting monolayer of water-soluble thiols, this gives the QDs the ability to be dried out from an aqueous solution and subsequently redissolved multiple times. The effect of multiple drying-redissolving cycles was studied here. When the QDs were in solution, their spectral properties were observed using absorption and emission spectroscopy. In one example, the initial synthesis of the CdSe QDs was performed in methanol rather than water. This was done so that there would be a minimal amount of heating, which could contribute to QD growth, during the rotary evaporation process (methanol's boiling point is 64.7°C). After the CdSe QDs were dried, they were redissolved in another soluble alcohol (ethanol, b.p. = 78.4°C). Once again, after the spectral properties of the CdSe QDs were examined, the CdSe QDs were dried again and then finally redissolved in water.

**Figure 4:**
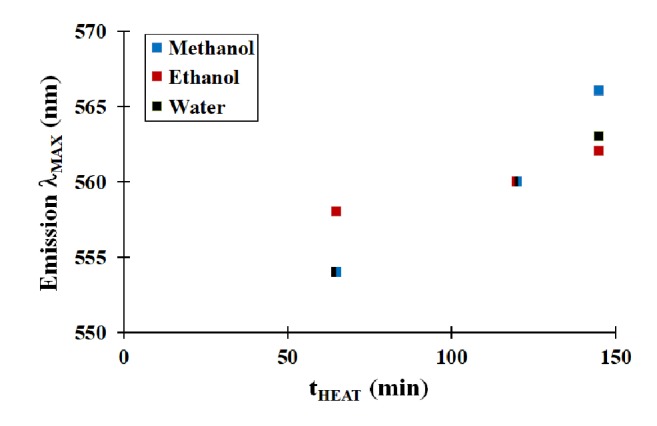
Effect of multiple drying-redissolving cycles on the fluorescence emission properties of mercaptosuccinic acid-modified CdSe QDs.

Figure 4 shows the wavelength at maximum absorbance (*λ*_MAX_) for several QD aliquots that underwent the aforementioned drying and redissolving cycles. To compare the effects of these cycles, one should focus on the three data points at a given heating time (t_HEAT_). When comparing the emission properties, it is clear that the QDs do not exhibit a significant difference in spectral properties at a particular t_HEAT_ value. Therefore, these nanomaterials can be dried and redissolved multiple times without any significant changes in their spectral properties.

### 3.4. Effect of Heating Temperature on Growth

Before QDs can be incorporated into potential applications, the growth of the nanoparticles needs to be controlled. The QDs prepared using the aqueous method are refluxed to increase the size of the nanoparticles. However, the QDs prepared in water tend to grow rather quickly during heating, which can be monitored by standard spectral techniques. The maximum emission or absorbance at a corresponding wavelength is a rough indicator of the QD size. In general, QDs that display shorter *λ*_MAX_ values tend to be smaller in size than those that have longer *λ*_MAX_ values. For example, the emission *λ*_MAX_ of the CdSe QD reaction mixture can transition from about 510 nm (solution color = yellow) to roughly 610 nm (red) in approximately 30 minutes (see Figure S2 for emission spectra of QDs). Slowing down this growth would allow for better tuning of the desired optical properties of the QDs.

**Figure 5:**
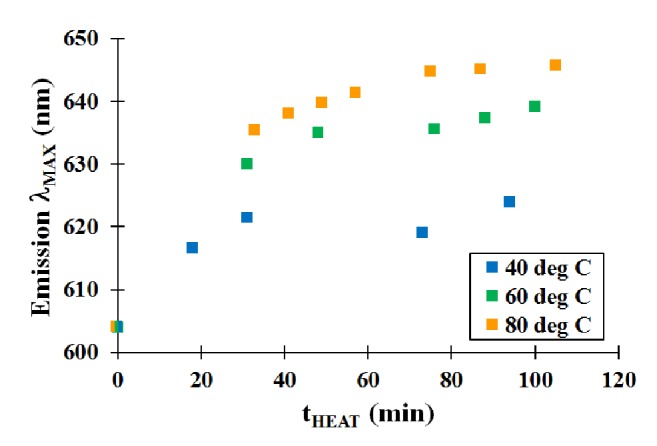
Effect of heating temperature on mercaptosuccinic acid-modified QDs.

One aspect that was studied was changing the heating temperature of the QD synthesis mixture. Typically, these materials were heated to reflux in water (100°C). Heating the QD mixture to reflux results in a rapid change in size based on the corresponding emission spectra, shifting to the red with heating time. To study the impact of heating temperature, the reaction mixture was heated to various temperatures (40°C, 60°C, and 80°C) over a two-hour period. Aliquots from the synthesis mixture were collected periodically, and their spectral properties were determined. As seen in Figure 5, there was a direct relationship between the speed of nanoparticle growth and temperature. Aqueous QDs grew most rapidly at 80°C, while those heated to 40°C exhibited slower growth. In each of the heating runs, the growth slows initially (near the onset of heating), then there are a short period of rapid growth and a gradual leveling off after about 40 minutes. This type of growth in semiconducting nanoparticles is similar to what was observed in previous studies [15, 16]. In most instances, after about one hour of heating, the growth appears to cease, with a shift to slightly more blue values for the maximum emission wavelength. This likely happens because of the aggregation of the nanoparticles over an extended heating time. Since more of the larger QD cores present coalesce, they precipitate out of the solution mixture, leaving smaller nanoparticles soluble in the aqueous solution. Since the smaller nanoparticles remain in solution, the QD sample would exhibit spectral properties that are slightly blue-shifted.

## 4. Conclusions

Water-soluble, monolayer-protected QDs were prepared using various cadmium salts. The use of these alternative cadmium salts did not have a significant impact on the spectral properties of the QDs. Water-soluble QDs were synthesized at low temperatures (< 0°C) using methanol and ethanol as reaction solvents instead of water. The synthesis at these lower temperatures resulted in slow nanoparticle growth upon subsequent heating of the reaction mixture. The change in solvent, from water to methanol or ethanol, had a significant effect on limiting the growth rate of the water-soluble QDs. Water-soluble QD samples that underwent several drying and redissolution cycles did not exhibit any significant changes in spectral properties. During the QD synthesis, increasing the heating temperature of the nanoparticle reaction mixture resulted in increased QD growth. In most instances, the nanoparticle growth profile can be described as starting with slow initial growth, and then there is a short period of rapid growth, reaching completion with an extended period of slow growth.

## Data Availability

The data used to support the findings of this study are available from the corresponding author upon request.

## Supplementary Materials

Supplementary Materials The supplementary material contains the datasets, as a spreadsheet file, to reproduce the results presented in this manuscript. Size-exclusion chromatography results and fluorescent spectra of QDs are also provided. Figure S1: fluorescence emission and electronic absorption (inset) properties of mercaptosuccinic acid-modified CdSe QDs separated using SEC. Figure S2: fluorescent spectra of glutathione-modified CdSe QDs synthesized in methanol at –35°C.
