# Supplementary material for: Limiting the Growth of Water-Soluble, Monolayer-Protected Quantum Dots
Source: Int J Anal Chem. 2018 Jul 2;2018:3164347. doi: 10.1155/2018/3164347 (PMC6051105; doi:10.1155/2018/3164347)
Supplement: Supplementary Materials — The supplementary material contains the datasets, as a spreadsheet file, to reproduce the results presented in this manuscript. Size-exclusion chromatography results and fluorescent spectra of QDs are also provided. Figure S1: fluorescence emission and electronic absorption (inset) properties of mercaptosuccinic acid-modified CdSe QDs separated using SEC. Figure S2: fluorescent spectra of glutathione-modified CdSe QDs synthesized in methanol at –35°C. [file 3164347.f1.zip › 3164347.f1.docx]

**Supplementary Materials**

**Limiting the growth of water-soluble, monolayer-protected quantum dots**

Ava E. Conner,^1^ Veronica Gordillo-Herrejon,^1^ Sonia C. Francone,^1^ Emily A. Shriner,^1^ Fernando E. Acosta,^1^ Nathan D. Barnett,^1^ and Deon T. Miles^1^

^1^ Chemistry Department, University of the South, Sewanee, TN 37383, United States.

Correspondence should be addressed to Deon T. Miles; dmiles@sewanee.edu

**Size exclusion chromatography (SEC)**

The ability to separate a polydisperse sample of nanoparticles into a more monodisperse one is a pursuit carried out by many nanoscientists.  Obtaining a sample of quantum dots with a narrow size distribution improves the spectral clarity of the nanomaterial.  Improved spectral clarity is important for certain applications using QDs.  Size exclusion chromatography (SEC) has been used to separate nanoparticles by size in a number of reports.^1^  While SEC is an established separation technique for biomolecules and polymers, its utility in separating large molecules has been translated into the field of nanomaterials. Here, QD samples were separated using a Sephadex G-75 SEC column.  Typically, smaller molecules are trapped in the pores while larger ones are not and therefore are eluted from the column faster. The eluted QD fractions are subsequently analyzed using common spectral techniques, as seen in Figure S1. With relation to fluorescence spectroscopy, smaller QDs emit shorter wavelengths of light compared to their larger counterparts. Therefore, those QD fractions that are eluted later, which are smaller in size, should emit at shorter wavelengths that earlier fractions.


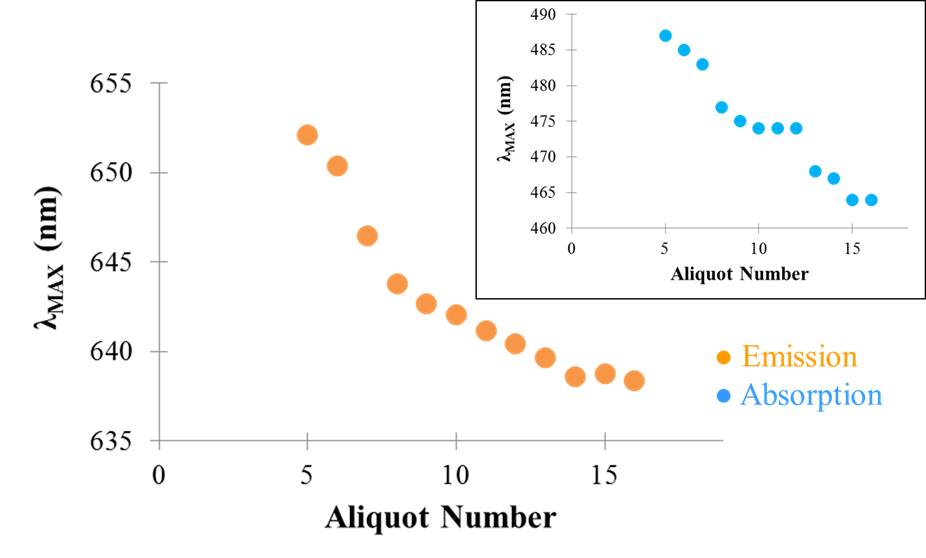


Figure S1: Fluorescence emission and electronic absorption (inset) properties of mercaptosuccinic acid-modified CdSe QDs separated using SEC.

**Low temperature synthesis**

An example of a typical low temperature synthesis is shown in Figure S2, where the temperature was held at –35 °C before the refluxing step.  Before heat was applied to the reaction mixture, the λ_MAX_ was about 505 nm.  After more than 24 hours of refluxing, the change in λ_MAX_ was roughly 45 nm.


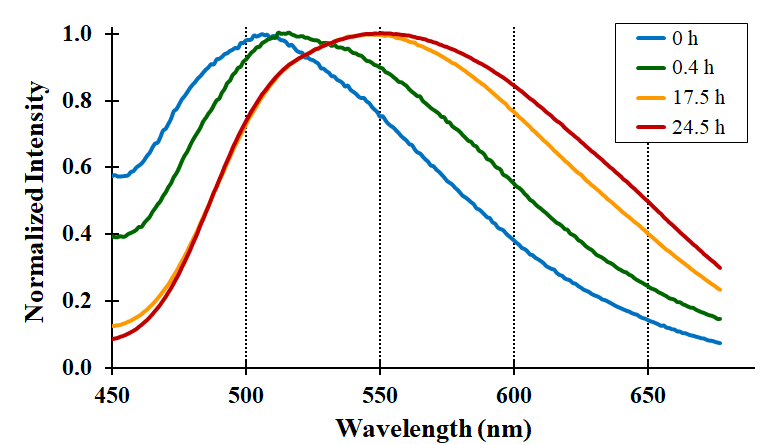


Figure S2: Fluorescent spectra of glutathione-modified CdSe QDs synthesized in methanol at –35 °C.
